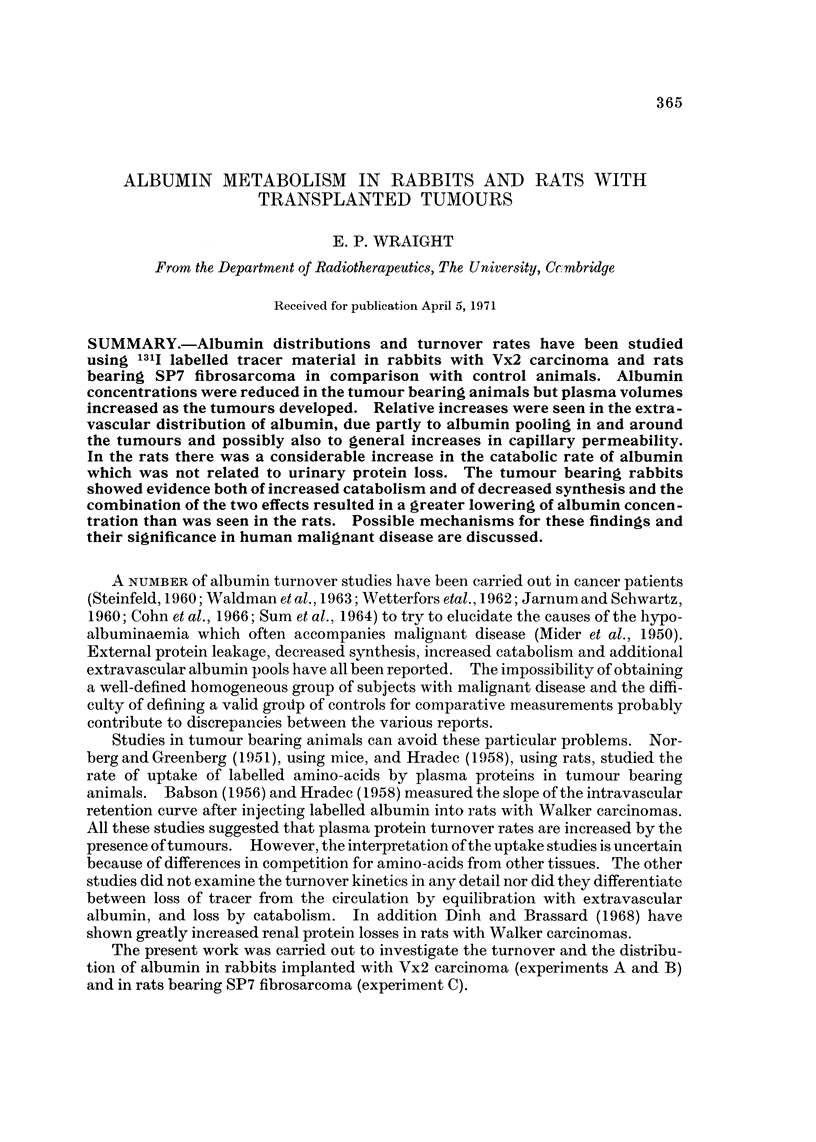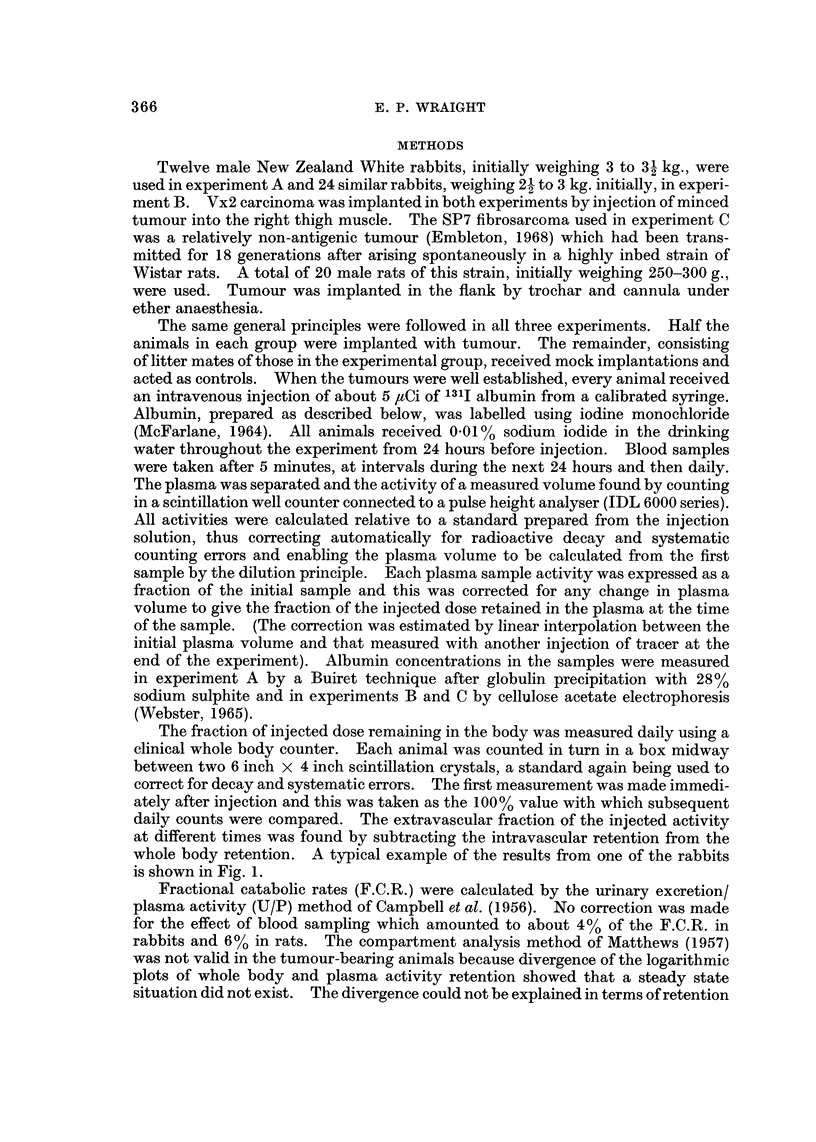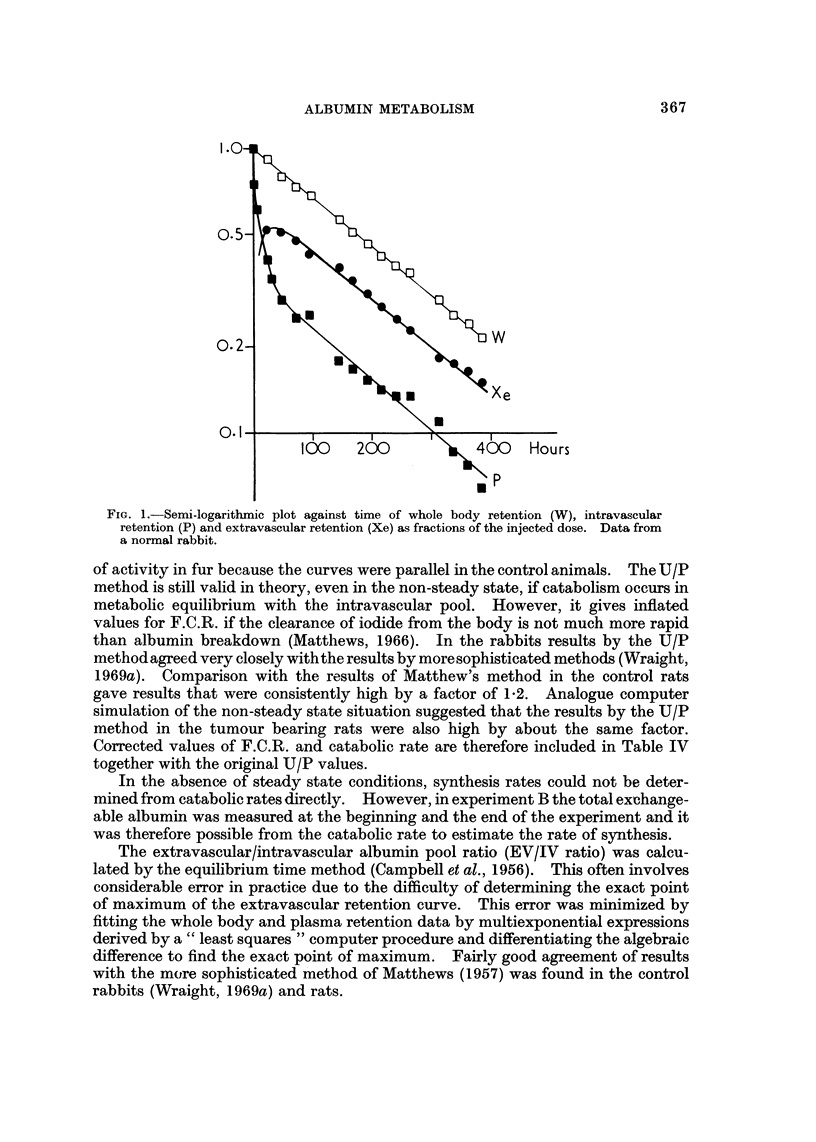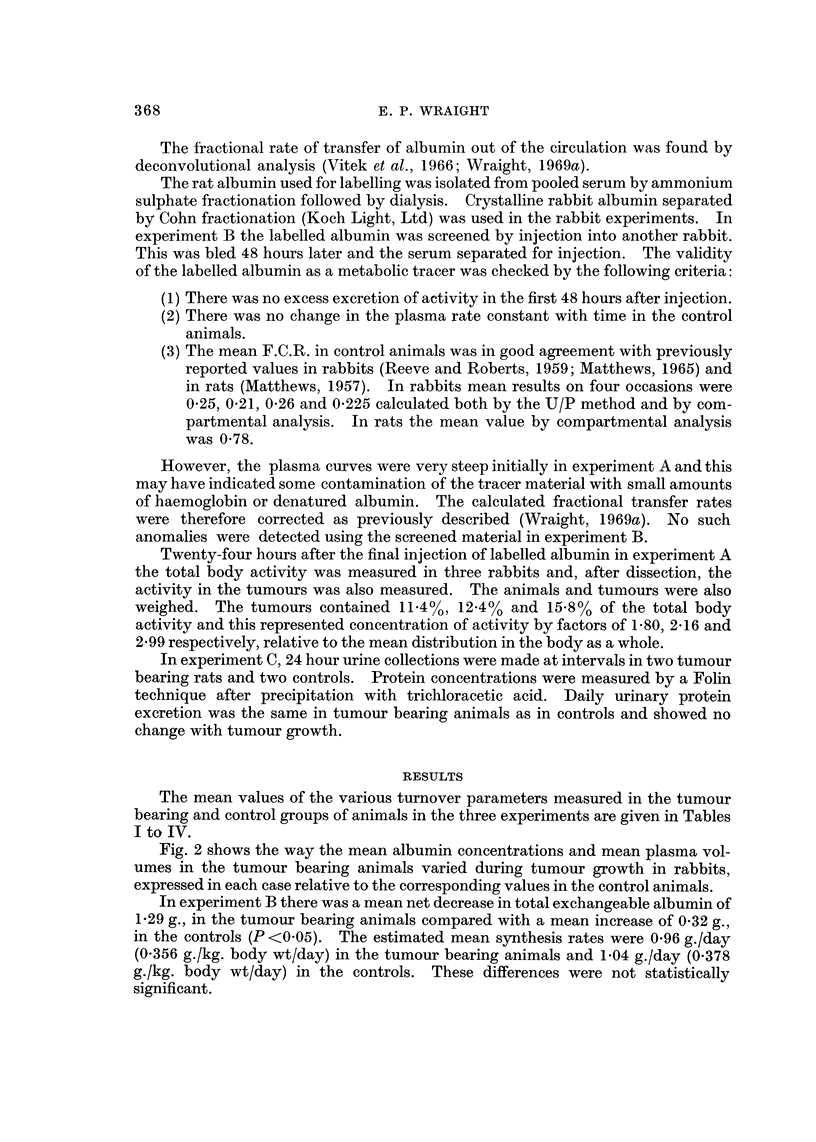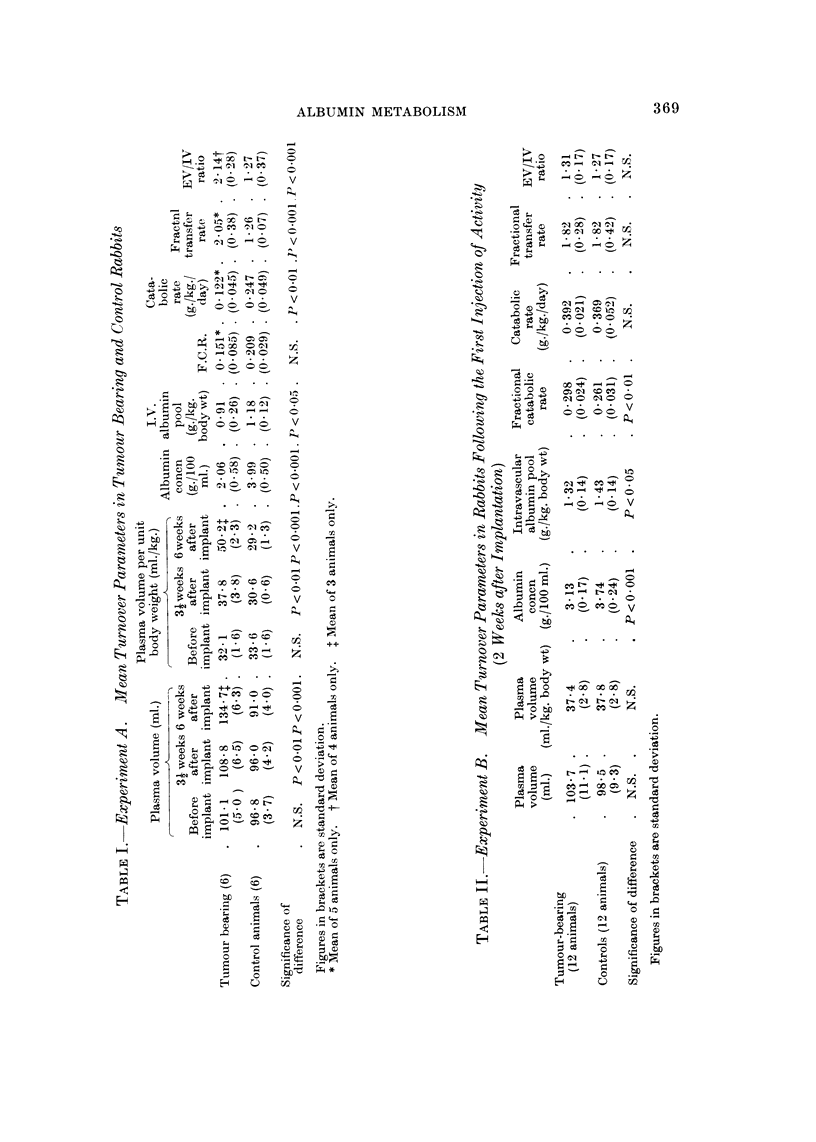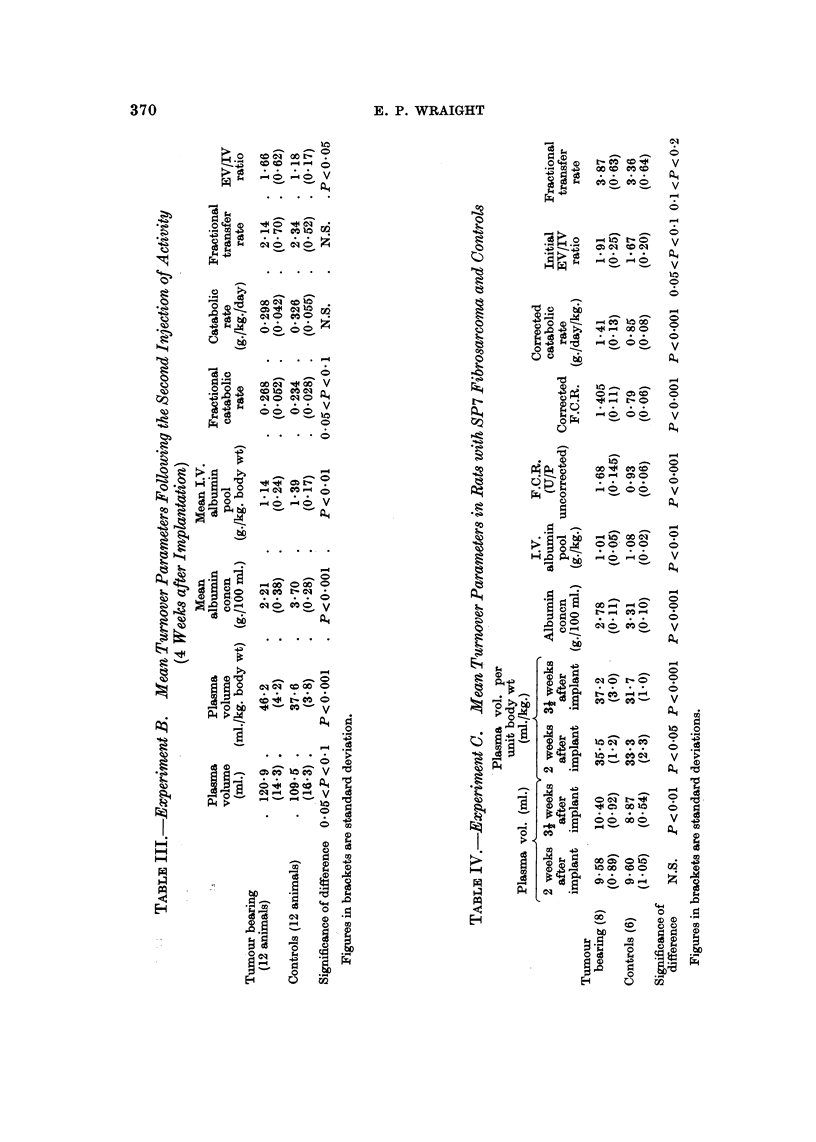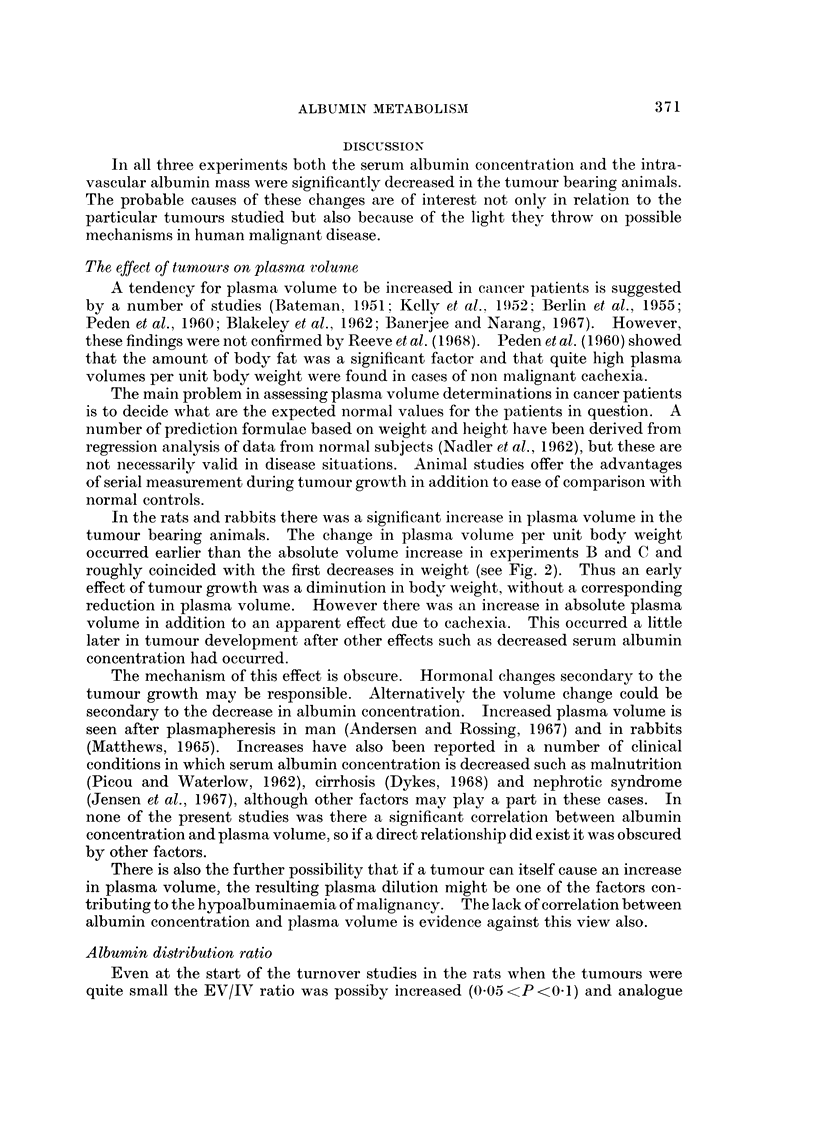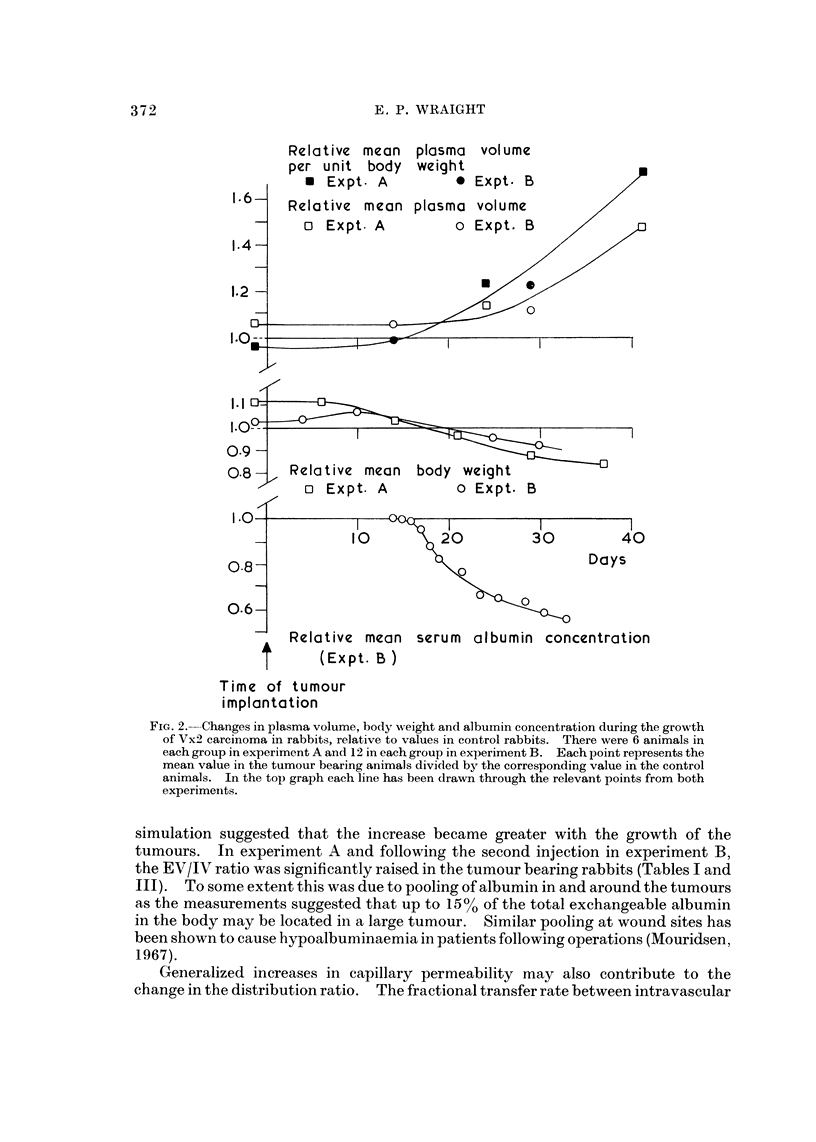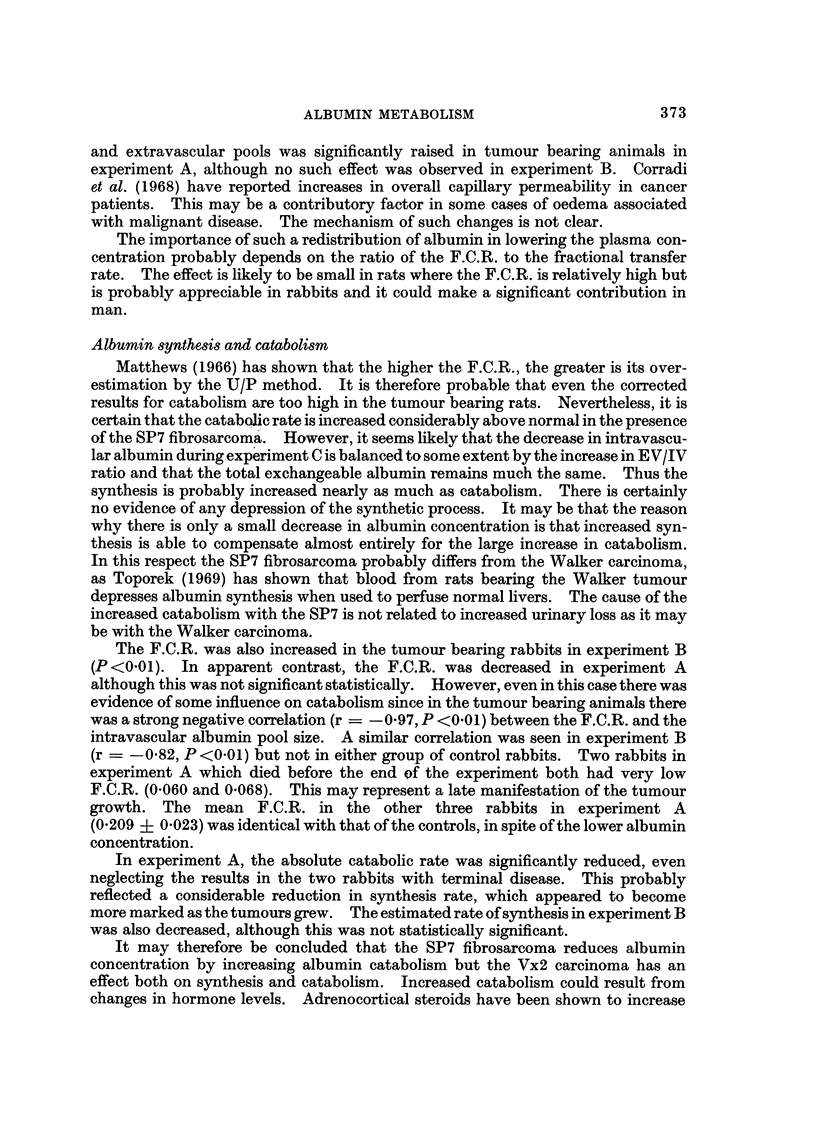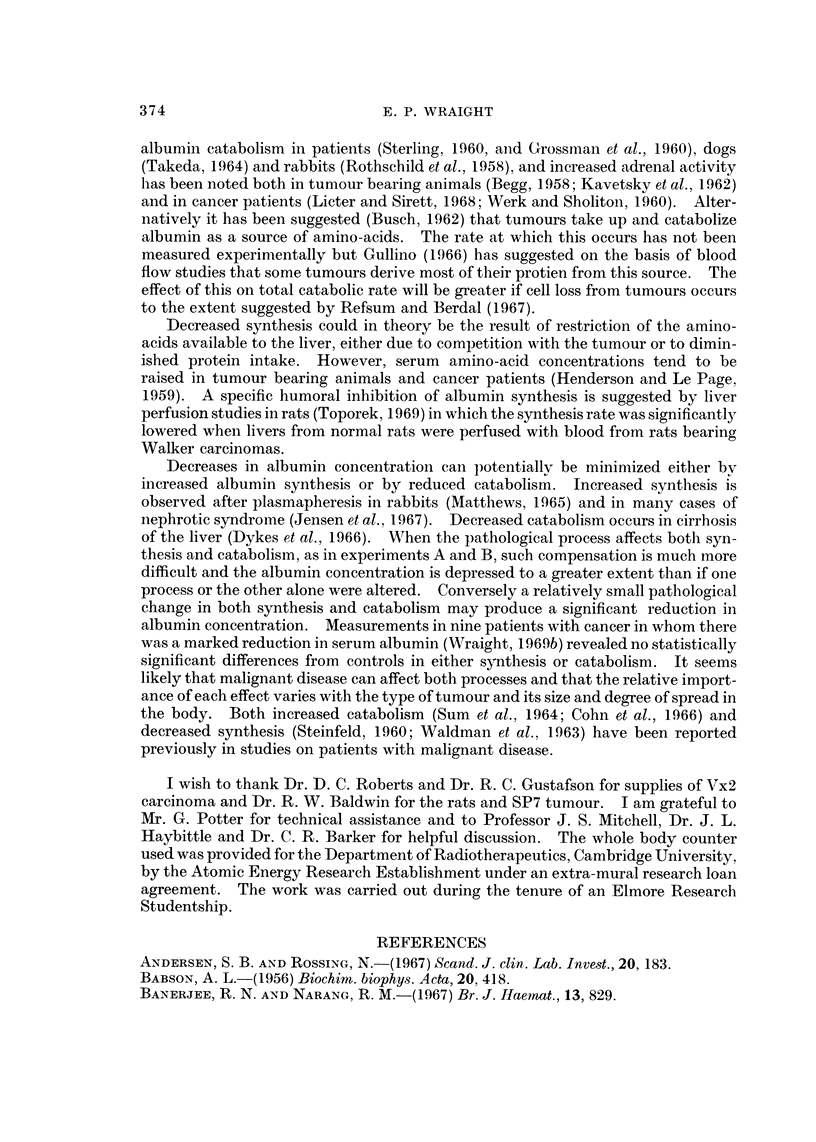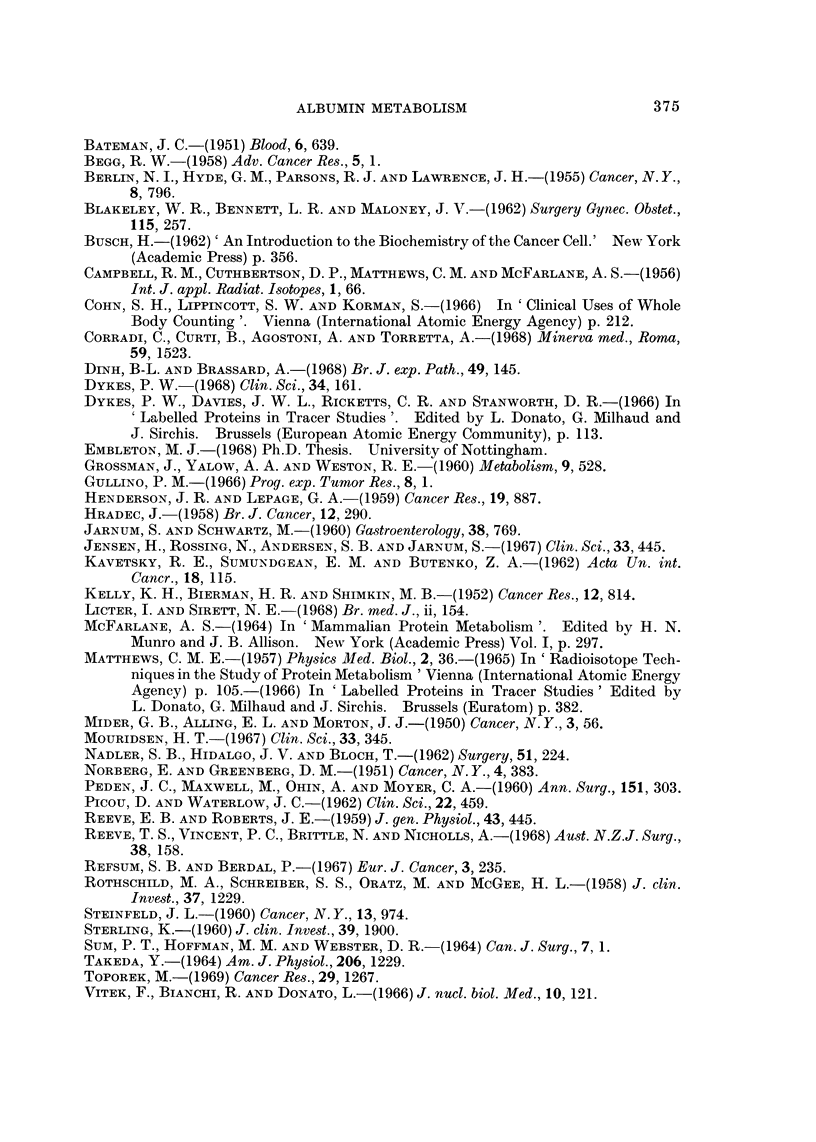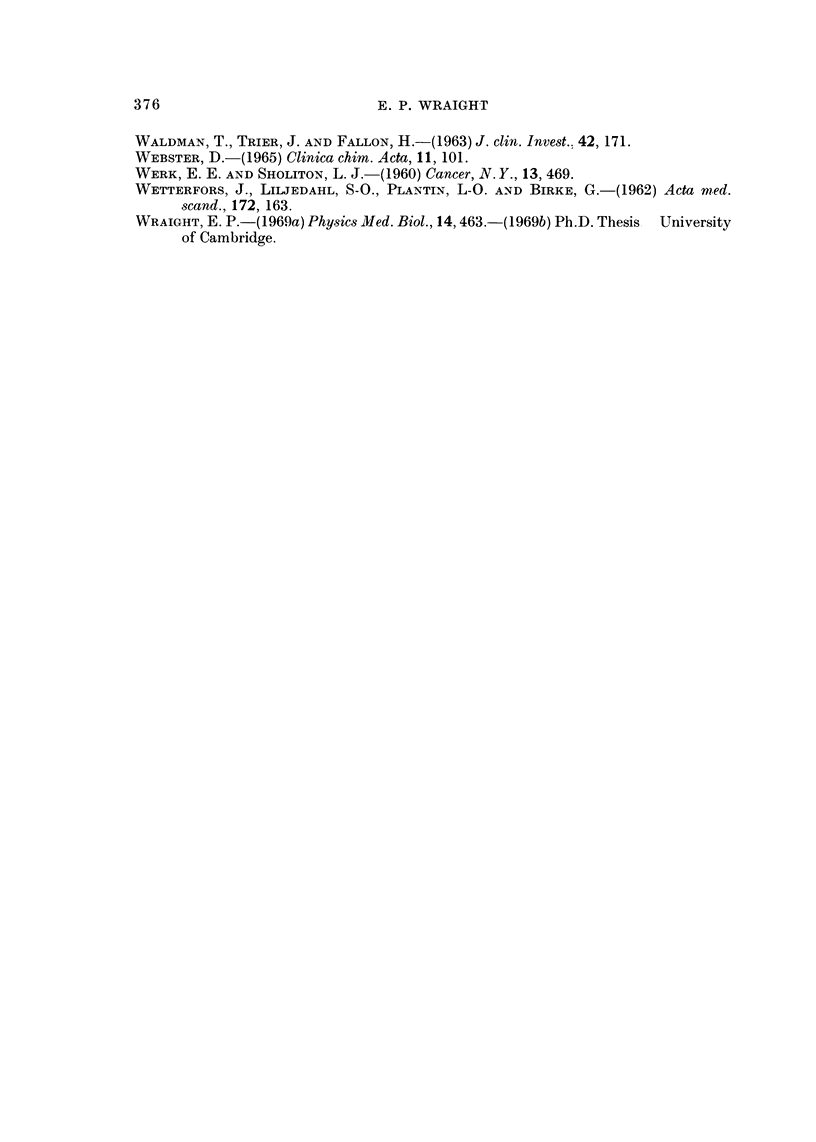# Albumin Metabolism in Rabbits and Rats with Transplanted Tumours

**DOI:** 10.1038/bjc.1971.47

**Published:** 1971-06

**Authors:** E. P. Wraight

## Abstract

Albumin distributions and turnover rates have been studied using ^131^I labelled tracer material in rabbits with Vx2 carcinoma and rats bearing SP7 fibrosarcoma in comparison with control animals. Albumin concentrations were reduced in the tumour bearing animals but plasma volumes increased as the tumours developed. Relative increases were seen in the extravascular distribution of albumin, due partly to albumin pooling in and around the tumours and possibly also to general increases in capillary permeability. In the rats there was a considerable increase in the catabolic rate of albumin which was not related to urinary protein loss. The tumour bearing rabbits showed evidence both of increased catabolism and of decreased synthesis and the combination of the two effects resulted in a greater lowering of albumin concentration than was seen in the rats. Possible mechanisms for these findings and their significance in human malignant disease are discussed.


### ALBUMIN METABOLISM IN RABBITS AND RATS WITH TRANSPLANTED TUMOURS

E. P. WRAIGHT

*From the Department of Radiotherapeutics, The University, Cambridge*

Received for publication April 5, 1971

**SUMMARY.—Albumin distributions and turnover rates have been studied using $^{131}I$ labelled tracer material in rabbits with Vx2 carcinoma and rats bearing SP7 fibrosarcoma in comparison with control animals. Albumin concentrations were reduced in the tumour bearing animals but plasma volumes increased as the tumours developed. Relative increases were seen in the extravascular distribution of albumin, due partly to albumin pooling in and around the tumours and possibly also to general increases in capillary permeability. In the rats there was a considerable increase in the catabolic rate of albumin which was not related to urinary protein loss. The tumour bearing rabbits showed evidence both of increased catabolism and of decreased synthesis and the combination of the two effects resulted in a greater lowering of albumin concentration than was seen in the rats. Possible mechanisms for these findings and their significance in human malignant disease are discussed.**

A NUMBER of albumin turnover studies have been carried out in cancer patients (Steinfeld, 1960; Waldman *et al.*, 1963; Wetterfors *et al.*, 1962; Jarnum and Schwartz, 1960; Cohn *et al.*, 1966; Sum *et al.*, 1964) to try to elucidate the causes of the hypoalbuminaemia which often accompanies malignant disease (Mider *et al.*, 1950). External protein leakage, decreased synthesis, increased catabolism and additional extravascular albumin pools have all been reported. The impossibility of obtaining a well-defined homogeneous group of subjects with malignant disease and the difficulty of defining a valid group of controls for comparative measurements probably contribute to discrepancies between the various reports.

Studies in tumour bearing animals can avoid these particular problems. Norberg and Greenberg (1951), using mice, and Hradec (1958), using rats, studied the rate of uptake of labelled amino-acids by plasma proteins in tumour bearing animals. Babson (1956) and Hradec (1958) measured the slope of the intravascular retention curve after injecting labelled albumin into rats with Walker carcinomas. All these studies suggested that plasma protein turnover rates are increased by the presence of tumours. However, the interpretation of the uptake studies is uncertain because of differences in competition for amino-acids from other tissues. The other studies did not examine the turnover kinetics in any detail nor did they differentiate between loss of tracer from the circulation by equilibration with extravascular albumin, and loss by catabolism. In addition Dinh and Brassard (1968) have shown greatly increased renal protein losses in rats with Walker carcinomas.

The present work was carried out to investigate the turnover and the distribution of albumin in rabbits implanted with Vx2 carcinoma (experiments A and B) and in rats bearing SP7 fibrosarcoma (experiment C).

### METHODS

Twelve male New Zealand White rabbits, initially weighing 3 to 3½ kg., were used in experiment A and 24 similar rabbits, weighing 2½ to 3 kg. initially, in experiment B. Vx2 carcinoma was implanted in both experiments by injection of minced tumour into the right thigh muscle. The SP7 fibrosarcoma used in experiment C was a relatively non-antigenic tumour (Embleton, 1968) which had been transmitted for 18 generations after arising spontaneously in a highly inbed strain of Wistar rats. A total of 20 male rats of this strain, initially weighing 250–300 g., were used. Tumour was implanted in the flank by trochar and cannula under ether anaesthesia.

The same general principles were followed in all three experiments. Half the animals in each group were implanted with tumour. The remainder, consisting of litter mates of those in the experimental group, received mock implantations and acted as controls. When the tumours were well established, every animal received an intravenous injection of about 5 $\mu$Ci of $^{131}$I albumin from a calibrated syringe. Albumin, prepared as described below, was labelled using iodine monochloride (McFarlane, 1964). All animals received 0·01% sodium iodide in the drinking water throughout the experiment from 24 hours before injection. Blood samples were taken after 5 minutes, at intervals during the next 24 hours and then daily. The plasma was separated and the activity of a measured volume found by counting in a scintillation well counter connected to a pulse height analyser (IDL 6000 series). All activities were calculated relative to a standard prepared from the injection solution, thus correcting automatically for radioactive decay and systematic counting errors and enabling the plasma volume to be calculated from the first sample by the dilution principle. Each plasma sample activity was expressed as a fraction of the initial sample and this was corrected for any change in plasma volume to give the fraction of the injected dose retained in the plasma at the time of the sample. (The correction was estimated by linear interpolation between the initial plasma volume and that measured with another injection of tracer at the end of the experiment). Albumin concentrations in the samples were measured in experiment A by a Buiret technique after globulin precipitation with 28% sodium sulphite and in experiments B and C by cellulose acetate electrophoresis (Webster, 1965).

The fraction of injected dose remaining in the body was measured daily using a clinical whole body counter. Each animal was counted in turn in a box midway between two 6 inch × 4 inch scintillation crystals, a standard again being used to correct for decay and systematic errors. The first measurement was made immediately after injection and this was taken as the 100% value with which subsequent daily counts were compared. The extravascular fraction of the injected activity at different times was found by subtracting the intravascular retention from the whole body retention. A typical example of the results from one of the rabbits is shown in Fig. 1.

Fractional catabolic rates (F.C.R.) were calculated by the urinary excretion/plasma activity (U/P) method of Campbell *et al.* (1956). No correction was made for the effect of blood sampling which amounted to about 4% of the F.C.R. in rabbits and 6% in rats. The compartment analysis method of Matthews (1957) was not valid in the tumour-bearing animals because divergence of the logarithmic plots of whole body and plasma activity retention showed that a steady state situation did not exist. The divergence could not be explained in terms of retention

Fig. 1.—Semi-logarithmic plot against time of whole body retention (W), intravascular retention (P) and extravascular retention (Xe) as fractions of the injected dose. Data from a normal rabbit.

of activity in fur because the curves were parallel in the control animals. The U/P method is still valid in theory, even in the non-steady state, if catabolism occurs in metabolic equilibrium with the intravascular pool. However, it gives inflated values for F.C.R. if the clearance of iodide from the body is not much more rapid than albumin breakdown (Matthews, 1966). In the rabbits results by the U/P method agreed very closely with the results by more sophisticated methods (Wraight, 1969*a*). Comparison with the results of Matthew's method in the control rats gave results that were consistently high by a factor of 1·2. Analogue computer simulation of the non-steady state situation suggested that the results by the U/P method in the tumour bearing rats were also high by about the same factor. Corrected values of F.C.R. and catabolic rate are therefore included in Table IV together with the original U/P values.

In the absence of steady state conditions, synthesis rates could not be determined from catabolic rates directly. However, in experiment B the total exchangeable albumin was measured at the beginning and the end of the experiment and it was therefore possible from the catabolic rate to estimate the rate of synthesis.

The extravascular/intravascular albumin pool ratio (EV/IV ratio) was calculated by the equilibrium time method (Campbell *et al.*, 1956). This often involves considerable error in practice due to the difficulty of determining the exact point of maximum of the extravascular retention curve. This error was minimized by fitting the whole body and plasma retention data by multiexponential expressions derived by a " least squares " computer procedure and differentiating the algebraic difference to find the exact point of maximum. Fairly good agreement of results with the more sophisticated method of Matthews (1957) was found in the control rabbits (Wraight, 1969*a*) and rats.

The fractional rate of transfer of albumin out of the circulation was found by deconvolutional analysis (Vitek *et al.*, 1966; Wraight, 1969*a*).

The rat albumin used for labelling was isolated from pooled serum by ammonium sulphate fractionation followed by dialysis. Crystalline rabbit albumin separated by Cohn fractionation (Koch Light, Ltd) was used in the rabbit experiments. In experiment B the labelled albumin was screened by injection into another rabbit. This was bled 48 hours later and the serum separated for injection. The validity of the labelled albumin as a metabolic tracer was checked by the following criteria:

(1) There was no excess excretion of activity in the first 48 hours after injection.
(2) There was no change in the plasma rate constant with time in the control animals.
(3) The mean F.C.R. in control animals was in good agreement with previously reported values in rabbits (Reeve and Roberts, 1959; Matthews, 1965) and in rats (Matthews, 1957). In rabbits mean results on four occasions were 0·25, 0·21, 0·26 and 0·225 calculated both by the U/P method and by compartmental analysis. In rats the mean value by compartmental analysis was 0·78.

However, the plasma curves were very steep initially in experiment A and this may have indicated some contamination of the tracer material with small amounts of haemoglobin or denatured albumin. The calculated fractional transfer rates were therefore corrected as previously described (Wraight, 1969*a*). No such anomalies were detected using the screened material in experiment B.

Twenty-four hours after the final injection of labelled albumin in experiment A the total body activity was measured in three rabbits and, after dissection, the activity in the tumours was also measured. The animals and tumours were also weighed. The tumours contained 11·4%, 12·4% and 15·8% of the total body activity and this represented concentration of activity by factors of 1·80, 2·16 and 2·99 respectively, relative to the mean distribution in the body as a whole.

In experiment C, 24 hour urine collections were made at intervals in two tumour bearing rats and two controls. Protein concentrations were measured by a Folin technique after precipitation with trichloracetic acid. Daily urinary protein excretion was the same in tumour bearing animals as in controls and showed no change with tumour growth.

### RESULTS

The mean values of the various turnover parameters measured in the tumour bearing and control groups of animals in the three experiments are given in Tables I to IV.

Fig. 2 shows the way the mean albumin concentrations and mean plasma volumes in the tumour bearing animals varied during tumour growth in rabbits, expressed in each case relative to the corresponding values in the control animals.

In experiment B there was a mean net decrease in total exchangeable albumin of 1·29 g., in the tumour bearing animals compared with a mean increase of 0·32 g., in the controls ($P < 0{\cdot}05$). The estimated mean synthesis rates were 0·96 g./day (0·356 g./kg. body wt/day) in the tumour bearing animals and 1·04 g./day (0·378 g./kg. body wt/day) in the controls. These differences were not statistically significant.

TABLE I.—*Experiment A. Mean Turnover Parameters in Tumour Bearing and Control Rabbits*

| | Plasma volume (ml.) | | | Plasma volume per unit body weight (ml./kg.) | | | Albumin concn (g./100 ml.) | I.V. albumin pool (g./kg. body wt) | F.C.R. | Catabolic rate (g./kg./day) | Fractnl transfer rate | EV/IV ratio |
|---|---|---|---|---|---|---|---|---|---|---|---|---|
| | Before implant | 3½ weeks after implant | 6 weeks after implant | Before implant | 3½ weeks after implant | 6 weeks after implant | | | | | | |
| Tumour bearing (6) | 101·1 (5·0 ) | 108·8 (6·5) | 134·7‡ (6·3) | 32·1 (1·6) | 37·8 (3·8) | 50·2‡ (2·3) | 2·06 (0·58) | 0·91 (0·26) | 0·151* (0·085) | 0·122* (0·045) | 2·05* (0·38) | 2·14† (0·28) |
| Control animals (6) | 96·8 (3·7) | 96·0 (4·2) | 91·0 (4·0) | 33·6 (1·6) | 30·6 (0·6) | 29·2 (1·3) | 3·99 (0·50) | 1·18 (0·12) | 0·209 (0·029) | 0·247 (0·049) | 1·26 (0·07) | 1·27 (0·37) |
| Significance of difference | N.S. | $P < 0·01$ | $P < 0·001$ | N.S. | $P < 0·01$ | $P < 0·001$ | $P < 0·001$ | $P < 0·05$ | N.S. | $P < 0·01$ | $P < 0·001$ | $P < 0·001$ |

Figures in brackets are standard deviation.
* Mean of 5 animals only. † Mean of 4 animals only. ‡ Mean of 3 animals only.

TABLE II.—*Experiment B. Mean Turnover Parameters in Rabbits Following the First Injection of Activity (2 Weeks after Implantation)*

| | Plasma volume (ml.) | Plasma volume (ml./kg. body wt) | Albumin concn (g./100 ml.) | Intravascular albumin pool (g./kg. body wt) | Fractional catabolic rate | Catabolic rate (g./kg./day) | Fractional transfer rate | EV/IV ratio |
|---|---|---|---|---|---|---|---|---|
| Tumour-bearing (12 animals) | 103·7 (11·1) | 37·4 (2·8) | 3·13 (0·17) | 1·32 (0·14) | 0·298 (0·024) | 0·392 (0·021) | 1·82 (0·28) | 1·31 (0·17) |
| Controls (12 animals) | 98·5 (9·3) | 37·8 (2·8) | 3·74 (0·24) | 1·43 (0·14) | 0·261 (0·031) | 0·369 (0·052) | 1·82 (0·42) | 1·27 (0·17) |
| Significance of difference | N.S. | N.S. | $P < 0·001$ | $P < 0·05$ | $P < 0·01$ | N.S. | N.S. | N.S. |

Figures in brackets are standard deviation.

TABLE III.—*Experiment B. Mean Turnover Parameters Following the Second Injection of Activity (4 Weeks after Implantation)*

| | Plasma volume (ml.) | Plasma volume (ml./kg. body wt) | Mean albumin concn (g./100 ml.) | Mean I.V. albumin pool (g./kg. body wt) | Fractional catabolic rate | Catabolic rate (g./kg./day) | Fractional transfer rate | EV/IV ratio |
|---|---|---|---|---|---|---|---|---|
| Tumour bearing (12 animals) | 120·9 | 46·2 | 2·21 | 1·14 | 0·268 | 0·298 | 2·14 | 1·66 |
| | (14·3) | (4·2) | (0·38) | (0·24) | (0·052) | (0·042) | (0·70) | (0·62) |
| Controls (12 animals) | 109·5 | 37·6 | 3·70 | 1·39 | 0·234 | 0·326 | 2·34 | 1·18 |
| | (16·3) | (3·8) | (0·28) | (0·17) | (0·028) | (0·055) | (0·52) | (0·17) |
| Significance of difference | $0{\cdot}05 < P < 0{\cdot}1$ | $P < 0{\cdot}001$ | $P < 0{\cdot}001$ | $P < 0{\cdot}01$ | $0{\cdot}05 < P < 0{\cdot}1$ | N.S. | N.S. | $P < 0{\cdot}05$ |

Figures in brackets are standard deviation.

TABLE IV.—*Experiment C. Mean Turnover Parameters in Rats with SP7 Fibrosarcoma and Controls*

| | Plasma vol. (ml.) | | Plasma vol. per unit body wt (ml./kg.) | | | | | | | | |
|---|---|---|---|---|---|---|---|---|---|---|---|
| | 2 weeks after implant | 3½ weeks after implant | 2 weeks after implant | 3½ weeks after implant | Albumin concn (g./100 ml.) | I.V. albumin pool (g./kg.) | F.C.R. (U/P uncorrected) | Corrected F.C.R. | Corrected catabolic rate (g./day/kg.) | Initial EV/IV ratio | Fractional transfer rate |
| Tumour bearing (8) | 9·58 | 10·40 | 35·5 | 37·2 | 2·78 | 1·01 | 1·68 | 1·405 | 1·41 | 1·91 | 3·87 |
| | (0·89) | (0·92) | (1·2) | (3·0) | (0·11) | (0·05) | (0·145) | (0·11) | (0·13) | (0·25) | (0·63) |
| Controls (6) | 9·60 | 8·87 | 33·3 | 31·7 | 3·31 | 1·08 | 0·93 | 0·79 | 0·85 | 1·67 | 3·36 |
| | (1·05) | (0·54) | (2·3) | (1·0) | (0·10) | (0·02) | (0·06) | (0·06) | (0·08) | (0·20) | (0·64) |
| Significance of difference | N.S. | $P < 0{\cdot}01$ | $P < 0{\cdot}05$ | $P < 0{\cdot}001$ | $P < 0{\cdot}001$ | $P < 0{\cdot}01$ | $P < 0{\cdot}001$ | $P < 0{\cdot}001$ | $P < 0{\cdot}001$ | $0{\cdot}05 < P < 0{\cdot}1$ | $0{\cdot}1 < P < 0{\cdot}2$ |

Figures in brackets are standard deviations.

### DISCUSSION

In all three experiments both the serum albumin concentration and the intravascular albumin mass were significantly decreased in the tumour bearing animals. The probable causes of these changes are of interest not only in relation to the particular tumours studied but also because of the light they throw on possible mechanisms in human malignant disease.

### *The effect of tumours on plasma volume*

A tendency for plasma volume to be increased in cancer patients is suggested by a number of studies (Bateman, 1951; Kelly *et al.*, 1952; Berlin *et al.*, 1955; Peden *et al.*, 1960; Blakeley *et al.*, 1962; Banerjee and Narang, 1967). However, these findings were not confirmed by Reeve *et al.* (1968). Peden *et al.* (1960) showed that the amount of body fat was a significant factor and that quite high plasma volumes per unit body weight were found in cases of non malignant cachexia.

The main problem in assessing plasma volume determinations in cancer patients is to decide what are the expected normal values for the patients in question. A number of prediction formulae based on weight and height have been derived from regression analysis of data from normal subjects (Nadler *et al.*, 1962), but these are not necessarily valid in disease situations. Animal studies offer the advantages of serial measurement during tumour growth in addition to ease of comparison with normal controls.

In the rats and rabbits there was a significant increase in plasma volume in the tumour bearing animals. The change in plasma volume per unit body weight occurred earlier than the absolute volume increase in experiments B and C and roughly coincided with the first decreases in weight (see Fig. 2). Thus an early effect of tumour growth was a diminution in body weight, without a corresponding reduction in plasma volume. However there was an increase in absolute plasma volume in addition to an apparent effect due to cachexia. This occurred a little later in tumour development after other effects such as decreased serum albumin concentration had occurred.

The mechanism of this effect is obscure. Hormonal changes secondary to the tumour growth may be responsible. Alternatively the volume change could be secondary to the decrease in albumin concentration. Increased plasma volume is seen after plasmapheresis in man (Andersen and Rossing, 1967) and in rabbits (Matthews, 1965). Increases have also been reported in a number of clinical conditions in which serum albumin concentration is decreased such as malnutrition (Picou and Waterlow, 1962), cirrhosis (Dykes, 1968) and nephrotic syndrome (Jensen *et al.*, 1967), although other factors may play a part in these cases. In none of the present studies was there a significant correlation between albumin concentration and plasma volume, so if a direct relationship did exist it was obscured by other factors.

There is also the further possibility that if a tumour can itself cause an increase in plasma volume, the resulting plasma dilution might be one of the factors contributing to the hypoalbuminaemia of malignancy. The lack of correlation between albumin concentration and plasma volume is evidence against this view also.

### *Albumin distribution ratio*

Even at the start of the turnover studies in the rats when the tumours were quite small the EV/IV ratio was possiby increased ($0{\cdot}05 < P < 0{\cdot}1$) and analogue

FIG. 2.—Changes in plasma volume, body weight and albumin concentration during the growth of Vx2 carcinoma in rabbits, relative to values in control rabbits. There were 6 animals in each group in experiment A and 12 in each group in experiment B. Each point represents the mean value in the tumour bearing animals divided by the corresponding value in the control animals. In the top graph each line has been drawn through the relevant points from both experiments.

simulation suggested that the increase became greater with the growth of the tumours. In experiment A and following the second injection in experiment B, the EV/IV ratio was significantly raised in the tumour bearing rabbits (Tables I and III). To some extent this was due to pooling of albumin in and around the tumours as the measurements suggested that up to 15% of the total exchangeable albumin in the body may be located in a large tumour. Similar pooling at wound sites has been shown to cause hypoalbuminaemia in patients following operations (Mouridsen, 1967).

Generalized increases in capillary permeability may also contribute to the change in the distribution ratio. The fractional transfer rate between intravascular and extravascular pools was significantly raised in tumour bearing animals in experiment A, although no such effect was observed in experiment B. Corradi *et al.* (1968) have reported increases in overall capillary permeability in cancer patients. This may be a contributory factor in some cases of oedema associated with malignant disease. The mechanism of such changes is not clear.

The importance of such a redistribution of albumin in lowering the plasma concentration probably depends on the ratio of the F.C.R. to the fractional transfer rate. The effect is likely to be small in rats where the F.C.R. is relatively high but is probably appreciable in rabbits and it could make a significant contribution in man.

*Albumin synthesis and catabolism*

Matthews (1966) has shown that the higher the F.C.R., the greater is its overestimation by the U/P method. It is therefore probable that even the corrected results for catabolism are too high in the tumour bearing rats. Nevertheless, it is certain that the catabolic rate is increased considerably above normal in the presence of the SP7 fibrosarcoma. However, it seems likely that the decrease in intravascular albumin during experiment C is balanced to some extent by the increase in EV/IV ratio and that the total exchangeable albumin remains much the same. Thus the synthesis is probably increased nearly as much as catabolism. There is certainly no evidence of any depression of the synthetic process. It may be that the reason why there is only a small decrease in albumin concentration is that increased synthesis is able to compensate almost entirely for the large increase in catabolism. In this respect the SP7 fibrosarcoma probably differs from the Walker carcinoma, as Toporek (1969) has shown that blood from rats bearing the Walker tumour depresses albumin synthesis when used to perfuse normal livers. The cause of the increased catabolism with the SP7 is not related to increased urinary loss as it may be with the Walker carcinoma.

The F.C.R. was also increased in the tumour bearing rabbits in experiment B ($P < 0{\cdot}01$). In apparent contrast, the F.C.R. was decreased in experiment A although this was not significant statistically. However, even in this case there was evidence of some influence on catabolism since in the tumour bearing animals there was a strong negative correlation ($r = -0{\cdot}97, P < 0{\cdot}01$) between the F.C.R. and the intravascular albumin pool size. A similar correlation was seen in experiment B ($r = -0{\cdot}82, P < 0{\cdot}01$) but not in either group of control rabbits. Two rabbits in experiment A which died before the end of the experiment both had very low F.C.R. (0·060 and 0·068). This may represent a late manifestation of the tumour growth. The mean F.C.R. in the other three rabbits in experiment A ($0{\cdot}209 \pm 0{\cdot}023$) was identical with that of the controls, in spite of the lower albumin concentration.

In experiment A, the absolute catabolic rate was significantly reduced, even neglecting the results in the two rabbits with terminal disease. This probably reflected a considerable reduction in synthesis rate, which appeared to become more marked as the tumours grew. The estimated rate of synthesis in experiment B was also decreased, although this was not statistically significant.

It may therefore be concluded that the SP7 fibrosarcoma reduces albumin concentration by increasing albumin catabolism but the Vx2 carcinoma has an effect both on synthesis and catabolism. Increased catabolism could result from changes in hormone levels. Adrenocortical steroids have been shown to increase albumin catabolism in patients (Sterling, 1960, and Grossman *et al.*, 1960), dogs (Takeda, 1964) and rabbits (Rothschild *et al.*, 1958), and increased adrenal activity has been noted both in tumour bearing animals (Begg, 1958; Kavetsky *et al.*, 1962) and in cancer patients (Licter and Sirett, 1968; Werk and Sholiton, 1960). Alternatively it has been suggested (Busch, 1962) that tumours take up and catabolize albumin as a source of amino-acids. The rate at which this occurs has not been measured experimentally but Gullino (1966) has suggested on the basis of blood flow studies that some tumours derive most of their protien from this source. The effect of this on total catabolic rate will be greater if cell loss from tumours occurs to the extent suggested by Refsum and Berdal (1967).

Decreased synthesis could in theory be the result of restriction of the amino-acids available to the liver, either due to competition with the tumour or to diminished protein intake. However, serum amino-acid concentrations tend to be raised in tumour bearing animals and cancer patients (Henderson and Le Page, 1959). A specific humoral inhibition of albumin synthesis is suggested by liver perfusion studies in rats (Toporek, 1969) in which the synthesis rate was significantly lowered when livers from normal rats were perfused with blood from rats bearing Walker carcinomas.

Decreases in albumin concentration can potentially be minimized either by increased albumin synthesis or by reduced catabolism. Increased synthesis is observed after plasmapheresis in rabbits (Matthews, 1965) and in many cases of nephrotic syndrome (Jensen *et al.*, 1967). Decreased catabolism occurs in cirrhosis of the liver (Dykes *et al.*, 1966). When the pathological process affects both synthesis and catabolism, as in experiments A and B, such compensation is much more difficult and the albumin concentration is depressed to a greater extent than if one process or the other alone were altered. Conversely a relatively small pathological change in both synthesis and catabolism may produce a significant reduction in albumin concentration. Measurements in nine patients with cancer in whom there was a marked reduction in serum albumin (Wraight, 1969*b*) revealed no statistically significant differences from controls in either synthesis or catabolism. It seems likely that malignant disease can affect both processes and that the relative importance of each effect varies with the type of tumour and its size and degree of spread in the body. Both increased catabolism (Sum *et al.*, 1964; Cohn *et al.*, 1966) and decreased synthesis (Steinfeld, 1960; Waldman *et al.*, 1963) have been reported previously in studies on patients with malignant disease.

I wish to thank Dr. D. C. Roberts and Dr. R. C. Gustafson for supplies of Vx2 carcinoma and Dr. R. W. Baldwin for the rats and SP7 tumour. I am grateful to Mr. G. Potter for technical assistance and to Professor J. S. Mitchell, Dr. J. L. Haybittle and Dr. C. R. Barker for helpful discussion. The whole body counter used was provided for the Department of Radiotherapeutics, Cambridge University, by the Atomic Energy Research Establishment under an extra-mural research loan agreement. The work was carried out during the tenure of an Elmore Research Studentship.

### REFERENCES

ANDERSEN, S. B. AND ROSSING, N.—(1967) *Scand. J. clin. Lab. Invest.*, **20**, 183.
BABSON, A. L.—(1956) *Biochim. biophys. Acta*, **20**, 418.
BANERJEE, R. N. AND NARANG, R. M.—(1967) *Br. J. Haemat.*, **13**, 829.

BATEMAN, J. C.—(1951) *Blood*, **6**, 639.
BEGG, R. W.—(1958) *Adv. Cancer Res.*, **5**, 1.
BERLIN, N. I., HYDE, G. M., PARSONS, R. J. AND LAWRENCE, J. H.—(1955) *Cancer, N.Y.*, **8**, 796.
BLAKELEY, W. R., BENNETT, L. R. AND MALONEY, J. V.—(1962) *Surgery Gynec. Obstet.*, **115**, 257.
BUSCH, H.—(1962) ' An Introduction to the Biochemistry of the Cancer Cell.' New York (Academic Press) p. 356.
CAMPBELL, R. M., CUTHBERTSON, D. P., MATTHEWS, C. M. AND MCFARLANE, A. S.—(1956) *Int. J. appl. Radiat. Isotopes*, **1**, 66.
COHN, S. H., LIPPINCOTT, S. W. AND KORMAN, S.—(1966) In ' Clinical Uses of Whole Body Counting '. Vienna (International Atomic Energy Agency) p. 212.
CORRADI, C., CURTI, B., AGOSTONI, A. AND TORRETTA, A.—(1968) *Minerva med., Roma*, **59**, 1523.
DINH, B-L. AND BRASSARD, A.—(1968) *Br. J. exp. Path.*, **49**, 145.
DYKES, P. W.—(1968) *Clin. Sci.*, **34**, 161.
DYKES, P. W., DAVIES, J. W. L., RICKETTS, C. R. AND STANWORTH, D. R.—(1966) In ' Labelled Proteins in Tracer Studies '. Edited by L. Donato, G. Milhaud and J. Sirchis. Brussels (European Atomic Energy Community), p. 113.
EMBLETON, M. J.—(1968) Ph.D. Thesis. University of Nottingham.
GROSSMAN, J., YALOW, A. A. AND WESTON, R. E.—(1960) *Metabolism*, **9**, 528.
GULLINO, P. M.—(1966) *Prog. exp. Tumor Res.*, **8**, 1.
HENDERSON, J. R. AND LEPAGE, G. A.—(1959) *Cancer Res.*, **19**, 887.
HRADEC, J.—(1958) *Br. J. Cancer*, **12**, 290.
JARNUM, S. AND SCHWARTZ, M.—(1960) *Gastroenterology*, **38**, 769.
JENSEN, H., ROSSING, N., ANDERSEN, S. B. AND JARNUM, S.—(1967) *Clin. Sci.*, **33**, 445.
KAVETSKY, R. E., SUMUNDGEAN, E. M. AND BUTENKO, Z. A.—(1962) *Acta Un. int. Cancr.*, **18**, 115.
KELLY, K. H., BIERMAN, H. R. AND SHIMKIN, M. B.—(1952) *Cancer Res.*, **12**, 814.
LICTER, I. AND SIRETT, N. E.—(1968) *Br. med. J.*, ii, 154.
MCFARLANE, A. S.—(1964) In ' Mammalian Protein Metabolism '. Edited by H. N. Munro and J. B. Allison. New York (Academic Press) Vol. I, p. 297.
MATTHEWS, C. M. E.—(1957) *Physics Med. Biol.*, **2**, 36.—(1965) In ' Radioisotope Techniques in the Study of Protein Metabolism ' Vienna (International Atomic Energy Agency) p. 105.—(1966) In ' Labelled Proteins in Tracer Studies ' Edited by L. Donato, G. Milhaud and J. Sirchis. Brussels (Euratom) p. 382.
MIDER, G. B., ALLING, E. L. AND MORTON, J. J.—(1950) *Cancer, N.Y.*, **3**, 56.
MOURIDSEN, H. T.—(1967) *Clin. Sci.*, **33**, 345.
NADLER, S. B., HIDALGO, J. V. AND BLOCH, T.—(1962) *Surgery*, **51**, 224.
NORBERG, E. AND GREENBERG, D. M.—(1951) *Cancer, N.Y.*, **4**, 383.
PEDEN, J. C., MAXWELL, M., OHIN, A. AND MOYER, C. A.—(1960) *Ann. Surg.*, **151**, 303.
PICOU, D. AND WATERLOW, J. C.—(1962) *Clin. Sci.*, **22**, 459.
REEVE, E. B. AND ROBERTS, J. E.—(1959) *J. gen. Physiol.*, **43**, 445.
REEVE, T. S., VINCENT, P. C., BRITTLE, N. AND NICHOLLS, A.—(1968) *Aust. N.Z.J. Surg.*, **38**, 158.
REFSUM, S. B. AND BERDAL, P.—(1967) *Eur. J. Cancer*, **3**, 235.
ROTHSCHILD, M. A., SCHREIBER, S. S., ORATZ, M. AND MCGEE, H. L.—(1958) *J. clin. Invest.*, **37**, 1229.
STEINFELD, J. L.—(1960) *Cancer, N.Y.*, **13**, 974.
STERLING, K.—(1960) *J. clin. Invest.*, **39**, 1900.
SUM, P. T., HOFFMAN, M. M. AND WEBSTER, D. R.—(1964) *Can. J. Surg.*, **7**, 1.
TAKEDA, Y.—(1964) *Am. J. Physiol.*, **206**, 1229.
TOPOREK, M.—(1969) *Cancer Res.*, **29**, 1267.
VITEK, F., BIANCHI, R. AND DONATO, L.—(1966) *J. nucl. biol. Med.*, **10**, 121.

WALDMAN, T., TRIER, J. AND FALLON, H.—(1963) *J. clin. Invest.*, **42**, 171.
WEBSTER, D.—(1965) *Clinica chim. Acta*, **11**, 101.
WERK, E. E. AND SHOLITON, L. J.—(1960) *Cancer, N.Y.*, **13**, 469.
WETTERFORS, J., LILJEDAHL, S-O., PLANTIN, L-O. AND BIRKE, G.—(1962) *Acta med. scand.*, **172**, 163.
WRAIGHT, E. P.—(1969*a*) *Physics Med. Biol.*, **14**, 463.—(1969*b*) Ph.D. Thesis University of Cambridge.